# Prevalence of single-nucleotide variants in twenty-five pharmacogenes from a Cuban sample cohort

**DOI:** 10.3389/fphar.2024.1467036

**Published:** 2024-09-27

**Authors:** Elizabeth Reyes-Reyes, José Alfredo Herrera-Isidrón, Elizabeth Cuétara-Lugo, Zhiv Shkedy, Dirk Valkenborg, Claudina Angela Pérez-Novo, Gisselle Fernández-Peña, Idania González-Pérez, Miguel David Fernández-Pérez, Wim Vanden-Berghe, Idania Rodeiro-Guerra

**Affiliations:** ^1^ Laboratory of Clinical Experimental Pharmacology, Teaching and Research Department, Institute of Oncology and Radiobiology (INOR), Havana, Cuba; ^2^ Laboratory of Pharmacology, Department of Pharmacology, Institute of Marine Sciences (ICIMAR), Havana, Cuba; ^3^ Institute of Material Science and Technology (IMRE), University of Havana, Havana, Cuba; ^4^ Academic Department, Cuban Institute of Ophthalmology “Ramon Pando Ferrer”, Havana, Cuba; ^5^ Research group Centre for Statistics, Faculty of Sciences, Data Science Institute, University of Hasselt, Hasselt, Belgium; ^6^ Laboratory of Cell Death Signaling, Department of Biomedical Sciences, University of Antwerp, Antwerp, Belgium; ^7^ Institute of Basic and Preclinical Sciences “Victoria de Girón”, Medical University of Havana (UCMH), Havana, Cuba

**Keywords:** genetic variants, single-nucleotide variants, pharmacogenetic, Cuban population, admixed population, precision medicine

## Abstract

**Introduction:**

The Cuban population is genetically diverse, and information on the prevalence of genetic variants is still limited. As complex admixture processes have occurred, we hypothesized that the frequency of pharmacogenetic variants and drug responses may vary within the country. The aims of the study were to describe the frequency distribution of 43 single-nucleotide variants (SNVs) from 25 genes of pharmacogenetic interest within the Cuba population and in relation to other populations, while taking into consideration some descriptive variables such as place of birth and skin color.

**Materials and Methods:**

SNVs were analyzed in 357 unrelated healthy Cuban volunteers. Genotype, allele frequencies, and ancestry proportions were determined, and the pairwise fixation index (F_ST_ ) was evaluated.

**Results:**

Hardy–Weinberg equilibrium (HWE) deviations in six loci (rs11572103, rs2740574, rs776746, rs3025039, rs861539, and rs1762429) were identified. Minor allele frequencies (MAFs) ranged from 0.00 to 0.15 for variants in genes encoding xenobiotic metabolizing enzymes. They also ranged from 0.01 to 0.21 for variants in DNA repair, growth factors, methyltransferase, and methyl-binding proteins, while they ranged from 0.04 to 0.27 for variants in the O-6-methylguanine-DNA methyltransferase enzyme. Moderate genetic divergence was observed upon comparison to Africans (F_ST_ = 0.071 and SD 0.079), with 19 markers exhibiting moderate-to-large genetic differentiation. The average European, African, and Amerindian ancestry proportions were 67.8%, 27.2%, and 5.3%, respectively. Ancestry proportions differed by skin color and birthplace for both African and European components, with the exception of the European component, which showed no significant difference between individuals from Western and Eastern regions. Meanwhile, the statistical significance varied in comparisons by skin color and birthplace within the Amerindian component. Low genetic divergence was observed across geographical regions. We identified 12 variants showing moderate-to-large differentiation between White/Black individuals.

**Conclusion:**

Altogether, our results may support national strategies for the introduction of pharmacogenetic tools in clinical practice, contributing to the development of precision medicine in Cuba.

## 1 Introduction

Single-nucleotide variants (SNVs) occur throughout the genome and constitute most of the human genetic diversity (Robert and Pelletier, 2018). These genomic variants can alter all steps of gene expression and protein functionality, ultimately modifying the effects of environmental exposures. In the context of drug responses and the safety of commonly prescribed drugs, SNVs are key factors accounting for interindividual variability. Therefore, characterization of populations in terms of the frequency distribution of SNVs relevant to pharmacogenetics provides a powerful approach for evaluating the suitability of its clinical applications in drug selection and dosage optimization, improving therapeutic efficacy while reducing adverse effects.

The American continent is highly heterogeneous and presents the second-greatest number of abundant region-specific alleles (i.e., that are common in one continent but absent in the rest of the world), reflecting their unique evolution (Fedorova et al., 2022). A study of the Consortium of the Ibero-American Network of Pharmacogenetics and Pharmacogenomics (CEIBA-RIBEF) showed that interethnic variability in clinically relevant drug metabolizing enzymes (*CYP2D6*, *CYP2C9*, and *CYP2C19*) in Latin America leads to a lack of correlation between the ‘‘predicted’’ enzyme metabolic capacity and the genotype (Naranjo et al., 2018). Differences in admixture history in Latin American populations have important implications for the frequency distribution of variants associated with drug absorption, distribution, metabolism, and excretion (ADME) responses between and within populations (Céspedes-Garro et al., 2016), making extrapolation of data not suitable. By characterizing the admixture proportions in Latin American countries and the distribution of ADME variants of pharmacogenetic relevance, it should be possible to share health policies and logistic solutions between similar populations. At the individual level, individual ancestry proportions may determine the probability of having a pharmacogenetic relevant genotype (Bonifaz-Pena et al., 2014). Thus, collectively studying SNVs across human populations is of great interest to develop precision medicine.

Cuba is the most populated country in the Caribbean and has a rich genetic heritage. The population is essentially the result of admixture between Spaniards, West Africans, and, to a lesser degree, native Amerindians who inhabited the island. Evidence from historical and anthropological studies indicates that native populations were drastically reduced to a few thousand people within 50 years after 1492 (Guanche, 2020). Studies on the Cuban population, using ancestry informative marker (AIM) panels purposely designed to reveal differences in allele frequencies between Native American, West African, and European populations, have confirmed the small contribution of the Amerindian component to the current Cuban genetic background (Cintado et al., 2009; Diaz-Horta et al., 2010; Marcheco-Teruel et al., 2014; Fortes-Lima et al., 2018). Spanish immigration took place during more than four centuries and consisted mainly of male individuals, who intensely mixed with other ethnic components of the Cuba population (i.e., native and African women). During the XX century, a marked tendency was observed among the Spanish immigrants to establish themselves in the Western–Central areas of the country compared to the eastern region (Guanche, 2020). As a result, insights into Cuban contemporary population and admixture dynamics show different ancestry proportions across provinces and regions of the country (Marcheco-Teruel et al., 2014; Fortes-Lima et al., 2018).

Comparisons to other populations have identified interethnic variability in *CYP2D6* and *CYP2C9* allele frequencies and metabolic phenotypes among Cubans, Spaniards (Llerena et al., 2014), and Nicaraguans (Llerena et al., 2012). In addition, there is evidence of geographic variation within the Cuban population in allele and genotype frequency of rs1045642 *MDR1* when skin color categories are considered (Rodeiro et al., 2022). As complex admixture processes have occurred, we hypothesized that the frequency of pharmacogenetic variants and drug responses may vary within the country. Understanding the genetic heterogeneity and admixture of Cubans between geographical regions should have important implications for the design and interpretation of clinical trials, the implementation of pharmacogenetic tools for drug prescription and dosage adjustment, and the extrapolation of data from other, more homogeneous populations. Despite the increasing number of studies describing genetic biomarkers in Cubans (Sotomayor-Lugo et al., 2022; Camacho et al., 2011; García et al., 2010), information about population allele frequencies from a representative sample of the Cuban population is still limited. In accordance, this study aimed to describe the distribution of 43 SNVs in 25 target pharmacogenes for the Cuban population and in relation to other populations. It was also intended to characterize the distribution of the SNVs by considering the descriptive variables such as place of birth and skin color. This knowledge should provide valuable data for (Robert and Pelletier, 2018) understanding the allele and genotype distribution of variants in clinically relevant pharmacogenes in the country (Fedorova et al., 2022), implementing personalized medicine approaches and pharmacogenetic testing in public health policies, and (Naranjo et al., 2018) establishing public health priorities across the country.

## 2 Materials and methods

### 2.1 General description of the sample

The study included 357 Cuban healthy volunteers older than 18 years; female volunteers accounted for 52.7% (n = 188) of the sample. Demographic variables such as skin color and place of birth were recorded. The ethnic classification was based on skin color self-perception, as defined in the last national census (Oficina Nacional de Estadísticas e Información. Centro de Estudios de Población y Desarrollo, 2016). According to this classification, the Cuban population may be clustered into three categories based on skin color: ‘Blanco’’ (white), ‘‘Mestizo’’ (admixed), and ‘‘Negro’’ (black) individuals. From the whole sample, 190 volunteers self-identified as White individuals (53.2%), 101 as admixed individuals (28.3%), and 66 as Black individuals (18.5%), and according to the place of birth, participants were grouped into Western (48.7%, n = 174), Center (13.7%. n = 49), and Eastern (37.5%, n = 134) regions.

### 2.2 Genotyping and estimation of ancestry proportions

Whole blood samples were obtained by venipuncture. Genomic DNA extraction was performed using the QIAGEN DNeasy^®^ Blood and Tissue Kit, following the manufacturer’s recommendations. A total of 41 SNVs (see Supplementary Table 1) and 34 AIMs (Phillips et al., 2012) (AIM panel details provided in Supplementary Table 2) were determined by targeted sequencing (amplicon sequencing method) on a HiSeq X Ten sequencer (Illumina platform) according to Illumina protocols. The *GSTM1* null and *GSTT1* null variants were analyzed using one-step multiplex real-time RT-PCR followed by high-resolution melting (HRM) curve analysis. The primers used were *GSTT1*-forward TTC​CTT​ACT​GGT​CCT​CAC​ATC​TC, *GSTT1*-reverse GGA​AAA​GGG​TAC​AGA​CTG​GGG​A, *GSTM1*-forward AAC​TCC​CTG​AAA​AGC​TAA​AGC, and *GSTM1*-reverse GTT​GGG​CTC​AAA​TAT​ACG​GTG​G. The DNA amplification protocol included an initial denaturation at 95°C for 10 min, followed by 35 cycles of denaturation at 95°C for 10 s, annealing at 62°C for 30 s, and extension at 72°C for 25 s for DNA polymerization. The program of the melting curve analysis consisted of 95°C for 10 s, 65°C for 1 min, and then ramping from 65°C to 95°C at a rate of 0.1°C/s.

Global ancestry proportions were determined using the program STRUCTURE v 2.3.4 (Pritchard et al., 2000). The runs consisted of 100,000 Markov Chain Monte Carlo steps after a burn-in period of length 50,000 with 20 replicates for a K-value of 3. The admixture and correlated allele frequencies models were applied, and we used prior specification of the population of origin of reference samples. The results were combined using the online version of CLUMPAK (available at http://clumpak.tau.ac.il.) (Kopelman et al., 2015). Individuals from the Human Genome Diversity Project (HGDP) (Bergström et al., 2020) and 1000 Genomes Project (Fairley et al., 2019) datasets were used as reference ancestral populations. Based on Cuban admixture, reference genotypes included 107 Europeans (Iberians IBS) and 405 Africans (YRI Yoruba, ESN Nigeria, MSL Sierra Leona, and GWD Gambia). HGDP populations consisted of 60 Spanish, 35 Mexicans (Pima and Maya), 22 Brazil (Karitiana and Suri), and 7 Colombia (Colombians) genotypes. These were obtained using the online tool SPSmart SNPforID 34-plex variability browser (http://spsmart.cesga.es/snpforid.php) (Amigo et al., 2008).

### 2.3 Statistical analyses

Allele and genotype frequencies were calculated. The chi square (χ2) test was used to check the Hardy–Weinberg equilibrium (HWE) and Fisher exact test (Fisher–Freeman–Halton format) (Freeman and Halton, 1951) to compare the genotype distribution among subgroups. Statistical differences in ancestry proportions were evaluated using Kruskal–Wallis (skin color and place of birth) analysis of variance, followed by *post hoc* Dunn’s test using a Bonferroni corrected alpha of 0.017 for multiple comparisons. Statistical significance was set at p-value < 0.05 in the two-sided test. Statistical analyses were carried out using the RStudio programming environment for data analysis and “stats” package version 4.1.2 (R Core Team, 2023).

Wright’s F_ST_ statistic was used as a metric to quantify genetic differentiation at SNVs across populations (Martin et al., 2023; Phan et al., 2020) and within the Cuban population. Pairwise variant-specific F_ST_ values were calculated as $=\frac{{\left(p1-p2\right)}^{2}}{\left(p1+p2\right)\left(2-p1-p2\right)}$ , where p1 and p2 denote the frequency of a given allele in populations 1 and 2, respectively. It was defined that F_ST_ values less than 0.05 represent low genetic divergence, values between 0.05 and 0.15 denote moderate divergence, F_ST_ values between 0.15 and –0.25 indicate large divergence, and F_ST_ values greater than 0.25 represent very large divergence (Chen et al., 2010).

## 3 Results

### 3.1 Prevalence of target single-nucleotide variants in pharmacogenes in the Cuban population

The rs35742686 (CYP2D6*3) and rs4986893 (CYP2C19*3) were not detected. Conversely, null variants in the glutathione S-transferase (GST) system, mu 1 (GSTM1*0) and theta 1 (GSTT1*0), were identified. The frequency of the *GSTM1* null genotype was 0.39, while that of the *GSTT1* null genotype was 0.18. The frequency of individuals with the concomitant deletion of *GSTM1* and *GSTT1* was 0.07. Genotype and allele frequencies of the remaining 39 SNVs are shown in Table 1.

**TABLE 1:** Genotype and allele frequencies of 39 SNVs in a sample of Cuban individuals.

*SNP*	Genotype frequency			Alternative allele (95% CI)	HWE χ2 p-value
	Reference homozygous	Heterozygous	Alternative homozygous		
Targeted SNVs in xenobiotic biotransformation enzymes and transporter genes					
rs1048943 T>C	0.85	0.14	0.01	0.08 (0.05–0.11)	0.21
rs1799814 G>T	0.91	0.09	0.00	0.04 (0.02–0.07)	0.38
rs1065852 G>A	0.68	0.28	0.04	0.18 (0.14–0.22)	0.48
rs28371706 G>A	0.90	0.10	0.01	0.05 (0.03–0.08)	0.32
rs11572103 T>A	0.90	0.09	0.01	0.06 (0.03–0.08)	**0.01**
rs10509681 T>C	0.82	0.17	0.02	0.10 (0.07–0.13)	0.06
rs1799853 C>T	0.81	0.18	0.01	0.10 (0.07–0.13)	0.83
rs1057910 A>C	0.93	0.06	0.01	0.03 (0.01–0.05)	0.33
rs2242480 C>T	0.47	0.40	0.13	0.33 (0.28–0.38)	0.05
rs2740574 T>C	0.63	0.29	0.08	0.23 (0.73–0.82)	**0.01**
rs776746 T>C	0.14	0.35	0.52	0.69 (0.64–0.74)	**0.01**
rs4244285 G>A	0.76	0.22	0.02	0.13 (0.10–0.17)	0.69
rs2234922 A>G	0.64	0.30	0.06	0.21 (0.16–0.25)	0.07
rs2066853 G>A	0.61	0.32	0.06	0.22 (0.18–0.27)	0.24
rs1045642 G>A	0.40	0.45	0.15	0.37 (0.32–0.42)	0.58
Targeted SNVs in DNA repair, growth factors, and methylation proteins					
rs140695 T>C	0.05	0.34	0.61	0.78 (0.73–0.82)	0.71
rs603097 G>A	0.01	0.23	0.75	0.87 (0.83–0.91)	0.67
rs11121832 T>C	0.09	0.39	0.53	0.72 (0.67–0.77)	0.46
rs16828708 A>G	0.51	0.40	0.10	0.29 (0.25–0.34)	0.43
rs2072408 A>G	0.04	0.36	0.60	0.78 (0.73–0.82)	0.54
rs4792953 T>C	0.15	0.43	0.42	0.63 (0.58–0.68)	0.19
rs7359598 T>C	0.21	0.47	0.32	0.56 (0.50–0.61)	0.31
rs3025039 C>T	0.79	0.19	0.03	0.12 (0.08–0.15)	**0.04**
rs712829 G>T	0.47	0.41	0.11	0.32 (0.24–0.41)	0.59
*rs1042522 G>C	0.15	0.43	0.41	0.63 (0.58–0.68)	0.21
rs13181 T>G	0.51	0.39	0.10	0.29 (0.24–0.34)	0.34
rs25487 T>C	0.08	0.39	0.53	0.73 (0.68–0.77)	0.92
rs861539 G>A	0.50	0.37	0.12	0.31 (0.26–0.36)	**0.02**
Targeted SNVs in the *MGMT* gene					
rs10764896 G>A	0.21	0.46	0.32	0.56 (0.50–0.61)	0.25
rs11016798 C>T	0.34	0.45	0.21	0.44 (0.38–0.49)	0.07
rs11016879 A>G	0.11	0.46	0.43	0.66 (0.61–0.71)	0.69
rs11016885 T>C	0.53	0.37	0.10	0.28 (0.24–0.33)	0.10
rs12259379 G>T	0.68	0.28	0.04	0.18 (0.14–0.22)	0.19
rs4751104 G>A	0.35	0.46	0.19	0.42 (0.37–0.47)	0.26
rs12763287 T>G	0.81	0.17	0.02	0.11 (0.07–0.14)	0.09
rs1762429 C>T	0.30	0.42	0.27	0.49 (0.43–0.54)	**0.00**
rs1762438 C>T	0.24	0.51	0.25	0.51 (0.45–0.56)	0.71
rs4751115 T>C	0.15	0.45	0.40	0.62 (0.57–0.68)	0.38
rs7068306 C>G	0.48	0.41	0.10	0.31 (0.26–0.36)	0.60

Genome Assembly GRCh38 (plus strand) (Martin et al., 2023). (95% CI), 95% confidence intervals; HWE, Hardy–Weinberg equilibrium; *TP53 gene is on the minus chromosomal strand, and rs1042522 is a G/C variant that can make the description prone to confusion. Here is annotated on the plus chromosomal strand: for rs1042522 is located on the minus chromosomal strand of the TP53 gene and it is a G/C variant. Here it is annotated on the plus chromosomal strand: the G allele on the plus strand (C allele on the minus or coding strand) stands for 72Pro. The C allele on the plus strand (G allele on the coding strand) corresponds to Arg72 (Whirl‐Carrillo et al., 2021). N = 357. The first section displays the frequencies of 15 SNVs in 10 genes (*AhR*, *CYP1A1*, *CYP2D6*, *CYP2C8*, *CYP2C9*, *CYP3A4*, *CYP3A5*, *CYP2C19*, *EPHX1*, and *ABCB1*). The second section displays 13 SNVs in genes encoding: DNA methylation enzymes (*MTHFR*, *MBD*, *and EZH1/2*), growth factors (*VEGF and EGFR*) and DNA repair (*TP53*, *ERCC2*, *XRCC1*, and *XRCC3*). The third section shows frequencies of 11 SNVs in the *MGMT* gene.

Significant (p < 0.05) values are highlighted in bold.

**FIGURE 1:**
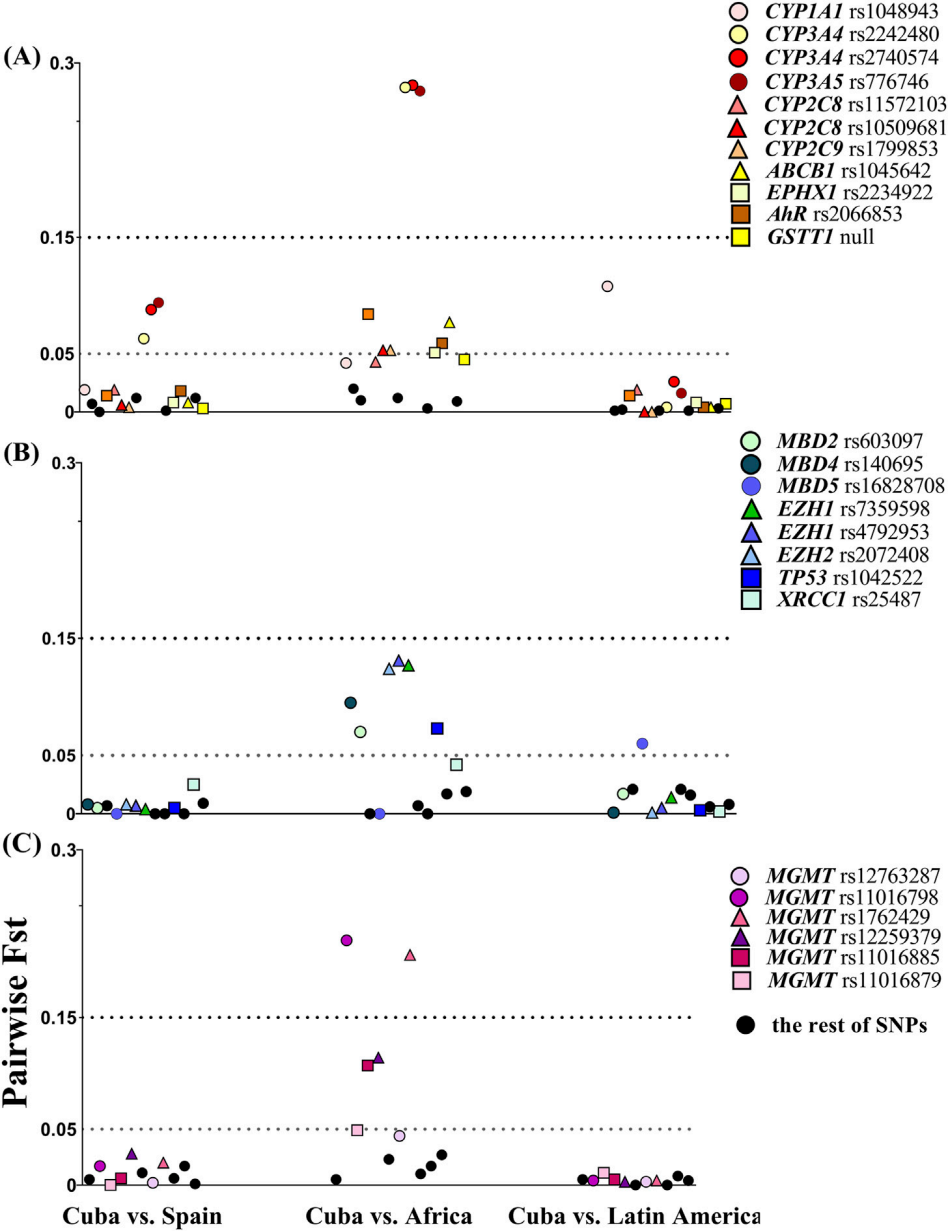
Allele-specific F_ST_ values in pairwise comparisons between Cuba and populations related by ancestry or geographical area. **(A)** Targeted SNVs in xenobiotic biotransformation enzymes and transporters. **(B)** Targeted SNVs in DNA repair, growth factors, methyltransferase, and methyl-binding proteins. **(C)** Targeted SNVs in the *MGMT* gene. The dashed lines show the F_ST_ threshold value at 0.05 for moderate divergence and 0.15 for large divergence. Frequency data from Latin America and Africa (Yoruba) were collected from the 1000 Genome Project database. Colored dots highlight loci that displayed small, moderate, and large genetic differentiation in pairwise comparisons with the Cuban population.

All SNVs, except for six variants (*CYP2C8* rs11572103, *CYP3A4* rs2740574, *CYP3A5* rs776746, *VEGF* rs3025039, *XRCC3* rs861539, and *MGMT* rs1762429), were in HWE. Population heterozygosity exhibited a wide range (6.0%–51.0%) among Cubans. Heterozygous genotypes were the most common genotype for *ABCB1* rs1045642, *EZH1* rs7359598, and SNVs in *MGMT*. In general terms, alternative homozygous genotypes in xenobiotic biotransformation enzymes showed low frequency (≤10.0%), with only three SNVs exhibiting frequencies higher than 10.0% (*CYP3A4* rs2242480, *CYP3A5* rs776746, and *ABCB1* rs1045642). Meanwhile, alternative homozygous genotypes were more frequent than the reference genotypes in *CYP3A5* rs776746, *MBD4* rs140695, *MBD2* rs603097, *MTHFR* rs11121832, *EZH2* rs2072408, and *XRCC1* rs25487. Alternative genotypes were usually higher than 10.0% in SNVs from the *MGMT* gene, with exceptions of 4.0% for rs12259379 and 2.0% for rs12763287. Afterward, the level of genetic differentiation between Cubans and other related populations was explored (Figure 1) (extended information is presented in Supplementary Table 3).

Pairwise F_ST_ analysis revealed that Cubans display high genetic similarity to Spanish and Latin American populations (Figure 1). Average pairwise F_ST_ values were 0.014 (SD 0.021) in Cuba vs. Spain and 0.011 (SD 0.019) for Cuba vs. Latin America comparisons. F_ST_ values were less than 0.05 for the majority of SNVs, indicating minimal genetic differentiation between these populations. However, few markers exceeded the F_ST_ threshold of moderate genetic differentiation (F_ST_ = 0.05), specifically *CYP3A4* and *CYP3A5* in Cuba vs. Spain and *CYP1A1* rs1048943 and *MBD5* in Cuba vs. Latin America.

Pairwise analysis for Cuba vs. Africa showed moderate divergence (mean F_ST_ = 0.071, SD 0.079). Variants in *CYP3A4*, *CYP3A5*, and *MGMT* (rs1762429 and rs11016798) presented large divergence (F_ST_ > 0.15), while other 14 SNVs in epigenetic proteins (*MBD2*, *MBD4*, *MBD5*, and *EZH1*/*2*), metabolic enzymes (*AHR*, *CYP2D6*, *CYP2C8*, *CYP2C9*, and *EPHX1*), and transport (*ABCB1*) and DNA repair-related proteins (*TP53* and *MGMT*) displayed moderate differentiation (Figure 1). Other variants with small differentiation nearly reached the moderate divergence threshold, namely, *CYP1A1* (rs1048943, F_ST_ = 0.042), *XRCC1* (F_ST_ = 0.042), *GSTT1* null (F_ST_ = 0.044), *CYP2C8* (rs11572103, F_ST_ = 0.042), and two SNVs in *MGMT* (rs12763287, F_ST_ = 0.044 and rs11016879, F_ST_ = 0.049).

### 3.2 Admixture proportions in the Cuban sample

**FIGURE 2:**
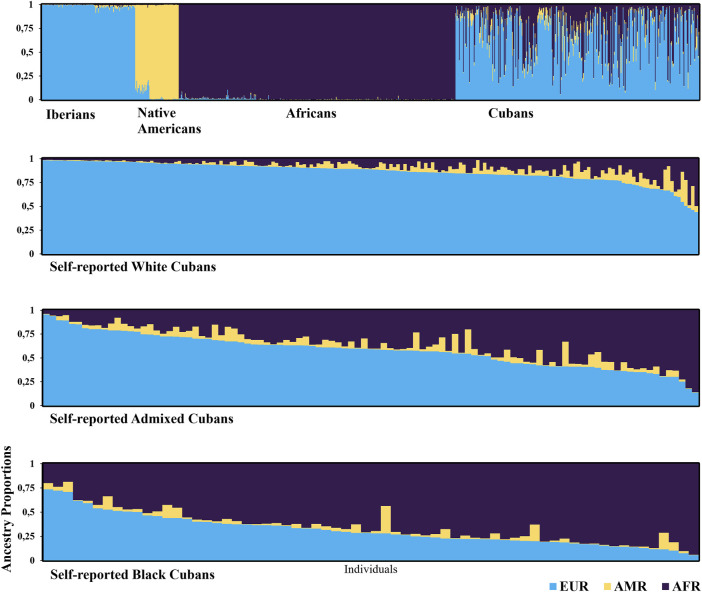
Estimated population structure using a genetic model-based clustering algorithm (STRUCTURE program). Each individual is represented by a vertical line, which is partitioned into colored segments that represent the individual’s estimated membership fractions in each cluster. Individuals can have membership in multiple clusters, with membership coefficients summing up to 1 in each cluster. Populations are labeled below each panel. The clusters are in different colors: blue for Europeans (EUR, Iberians), orange for Amerindians (AMR, Native Americans), and purple for Africans (AFR). Population data were collected from the databases HGDP-CEPH and 1000 Genome Project (n = 993): Cubans (n = 357), White Cubans (n = 190), Admixed Cubans (n = 101), and Black Cubans (n = 66).

The extent of admixture in the Cuban sample is illustrated in Figure 2 along with data from reference ancestral populations. There were clear differences in individual admixture estimates among these four population samples. The Cuban population was much more diverse in its ancestry proportions, and on average, individuals had large degrees of European (67.8%) and African ancestry (27.2%), while the contribution of Amerindian ancestry was small (5.0%). Average proportions of European ancestry decreased progressively among self-reported groups: White 85.9% (98.2–41.3), admixed 57.7% (95.5–13.6), and Black individuals 31.2% (73.6–5.4) (Kruskal–Wallis, KW: European, p < 0.001, followed by *post hoc* Dunn’s test). The opposite trend was observed in the African ancestry, which, respectively, averaged 9.3% (55.4–1.2), 36.3% (85.9–3.7), and 64.7% (94.1–18.7) in self-identified White, admixed, and Black individuals (KW: African, p < 0.001). Amerindian ancestry contribution was small across the three groups (KW, *p* = 0.004). *Post hoc* analysis indicated that Amerindian ancestry in admixed individuals (6.0%) was significantly different from that in self-identified White (4.8%) or Black individuals (4.1%).

The ancestry composition by place of birth showed distinctive geographic trends in admixture proportions. The Western and Central regions had higher European ancestry (70.4% and 83.3%, respectively) than the Eastern region (58.9%) (KW, *p* < 0.001). For African ancestry, the Eastern region showed larger proportions (34.1%), followed by the Western (26.9%) and the Central regions (8.9%), with significant differences among groups (KW, *p* < 0.001). Similar proportions of the Amerindian component were found in Central and Eastern regions (7.7% and 7.0%, respectively), but these were significantly different from the Western region (2.7%, KW, *p* < 0.001).

**FIGURE 3:**
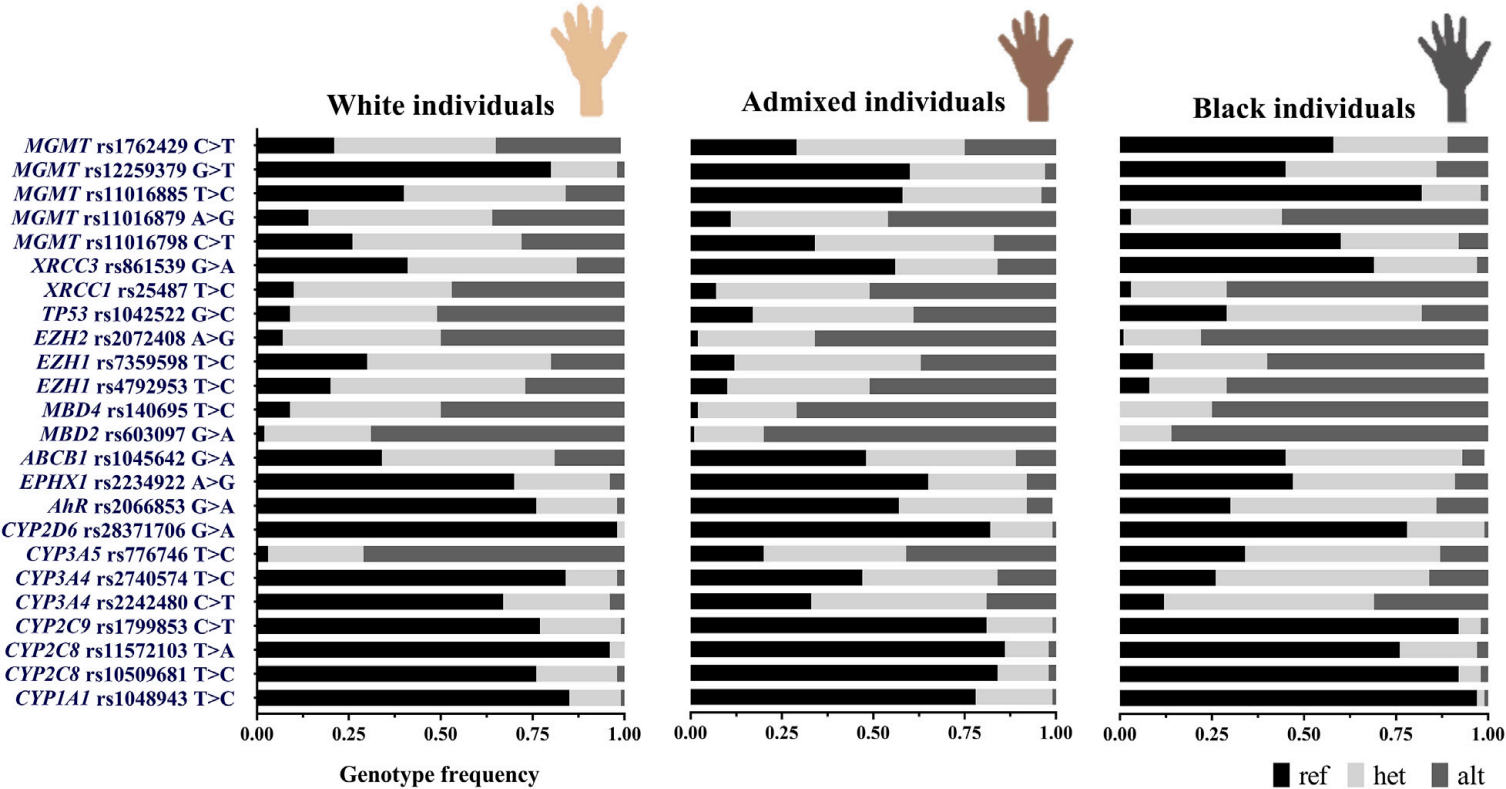
Distribution of 24 SNV genotype frequencies significantly different in the stratification analysis by individuals’ skin color (Fisher exact test), ref: reference homozygous genotype, het: heterozygous genotype, and alt: alternative homozygous genotype (N = 357, white = 190, admixed = 101, and black = 66).

Heterogeneous admixture levels were observed in individuals when skin color and place of birth were taken into consideration. In order to evaluate whether these variables could be useful to expose the distinctive distribution of the SNVs, we compared the genotype and allele frequencies and characterized the genetic differentiation using the F_ST_ statistic among subgroups. No significant differences were detected in allele frequencies across skin color subgroups or birthplace. However, genotype frequency significantly differed (Fisher exact test, *p* < 0.05) at 24 loci among skin color subgroups (Figure 3) and 11 loci among birthplaces. Extended information on the minor allele frequency (MAF) of the SNVs in this study compared to other populations is presented in Supplementary Table 4.bj

It can be noted that self-identified admixed individuals showed intermediate frequencies of the alternative genotypes compared to White/Black individuals. Admixed resembled Black individuals in the frequencies of five SNVs: *CYP3A4* rs2740574, *EPHX1*, *MBD2*, *MBD4*, and *MGMT* rs11016885, and they resembled White subjects in the frequencies of just two SNVs: *XRCC1* and *MGMT* rs12259379. Comparable alternative genotype frequencies between groups were observed for five SNVs: *CYP2D6* rs28371706, *CYP2C9*, *CYP2C8* rs11572103, rs10509681, and *CYP1A1* (Figure 3).

On the other hand, the frequencies of alternative genotypes were generally higher, either in the Central or Eastern regions, whereas the Western region showed intermediate frequencies. Particularly, alternative genotypes of the SNVs in *MGMT* rs1762429, rs11016798, *XRCC3*, *TP53*, and *CYP3A5* were predominant in the Central region, whereas SNVs in *EZH1*, *MBD4*, and *CYP3A4* rs2242480 had higher frequencies in the Eastern region. Only, the *EGFR* SNV showed the highest frequency in Western provinces (0.19), followed the Central provinces (0.08), and was not detected in the Eastern region.

**FIGURE 4:**
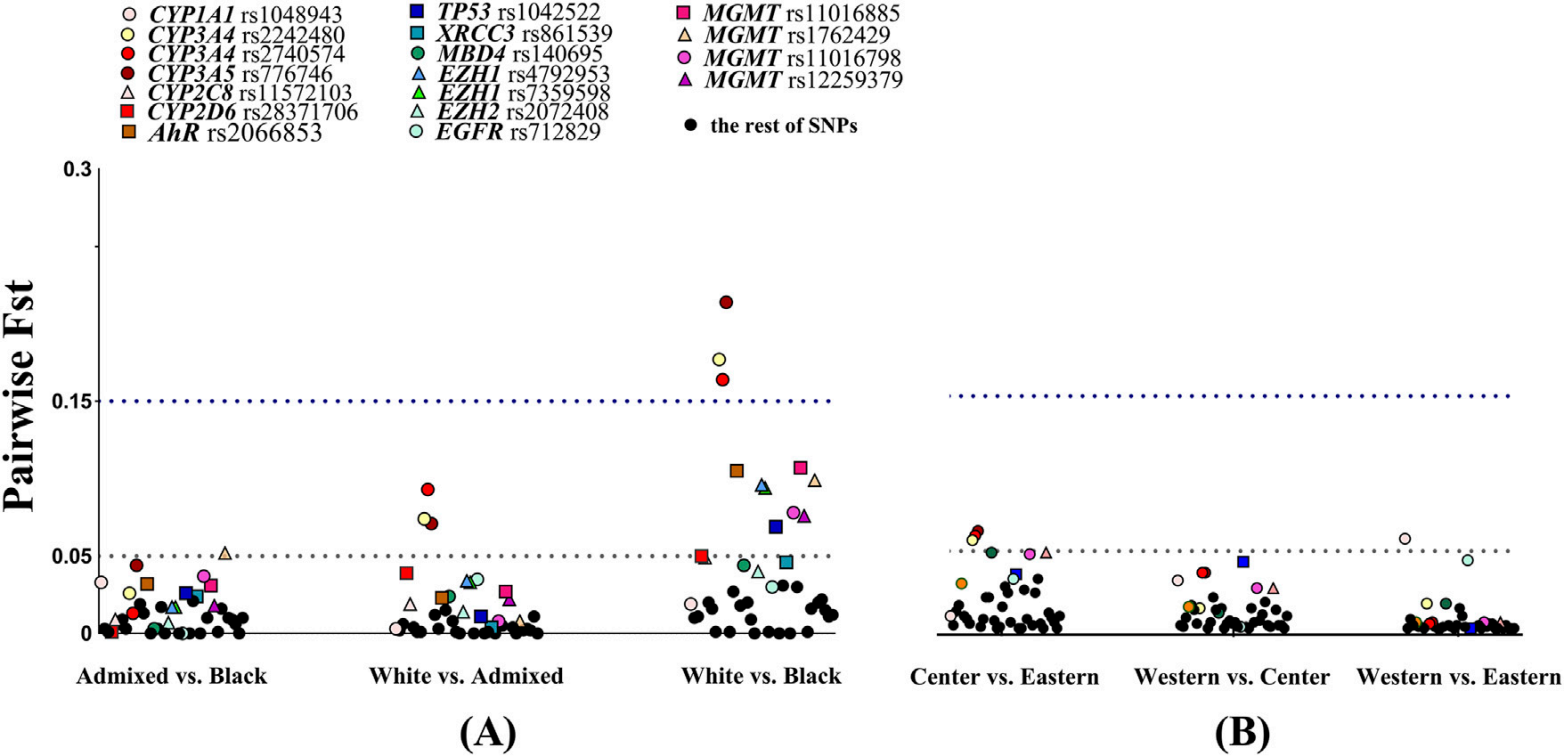
Allele-specific F_ST_ values for 41 variants in pairwise comparisons. **(A)** Stratified by skin color of individuals. **(B)** Stratified by region of origin. The dashed lines show the F_ST_ threshold at 0.05 for moderate divergence and 0.15 for large divergence. Color points specify SNVs with small, moderate, and large divergence in pairwise comparisons. N = 357, self-reported White n = 190, admixed n = 101, Black n = 66, Western n = 174, Central n = 49, and Eastern n = 134.

Genetic heterogeneity within the Cuban sample was explored using the F_ST_ statistic. Allele-specific pairwise F_ST_ analysis was conducted among skin color categories and geographical place of birth (Figure 4; Supplementary Table 3). Low genetic divergence was found when comparing self-identified admixed vs. Black and admixed vs. White individuals, with mean F_ST_ values of 0.013 (SD 0.013) and 0.015 (SD 0.022), respectively. The mean F_ST_ value of 0.045 (SD 0.051) indicated greater differentiation between self-identified White vs. Black individuals (Figure 4A). The variants in *CYP3A4* and *CYP3A5* genes showed moderate differentiation in White vs. admixed individuals but large differentiation in White vs. Black Cubans. Only *MGMT* rs1762429 (F_ST_ = 0.052) and *CYP3A5* (F_ST_ = 0.044) approached the F_ST_ threshold value for moderate genetic divergence in admixed vs. Black individuals. These results agreed with the admixture proportions (according to skin color) reported here. Variants in *AHR*, *TP53*, *EZH1*, and *CYP2D6* rs28371706 and four variants in *MGMT* displayed moderate divergence in White vs. Black Cubans, while *MBD4* (F_ST_ = 0.044), *CYP2C8* rs11572103 (F_ST_ = 0.049), and *XRCC3* (F_ST_ = 0.046) approached the moderate threshold.

Low genetic divergence was found between the Central vs. Eastern (F_ST_ = 0.017; SD = 0.019), Western vs. Central (F_ST_ = 0.010; SD = 0.011), and Western vs. Eastern (F_ST_ = 0.005; SD = 0.011) geographical regions (Figure 4B). Three SNVs, namely, *CYP3A4* rs2242480, rs2740574, and *CYP3A5* rs776746, exhibited moderate differentiation when Center vs. Eastern were compared and in *CYP1A1* rs1048943 for Western vs. Eastern regions.

Other SNVs were close to the moderate threshold: in *MBD4* (F_ST_ = 0.049) and two variants in *MGMT* when the Central vs. Eastern regions were compared. Meanwhile, *TP53* (F_ST_ = 0.043) in Western vs. Central and *EGFR* (F_ST_ = 0.044) in Western vs. Eastern regions were also close to the moderate threshold. In short, 12 SNVs surpassed the moderate threshold F_ST_ value when White vs. Black individuals were compared, while low divergence was observed in the rest of the comparisons.

## 4 Discussion

In this section, we studied the frequencies of 43 variants in genes affecting drug metabolism, transport, and pharmacological efficacy in healthy Cuban volunteers. Five out of the six SNVs that showed HWE deviations (Table 1) displayed statistical differences by skin color and four by birthplace. Except for *VEGF* rs3025039, the markers presented moderate-to-large genetic differentiation in pairwise comparisons within Cubans and with other populations. Hence, population stratification seems to be the most plausible explanation for the observed HWE deviations.

Ancestry analyses in this study revealed admixture diversity among individuals and within group of individuals from the same birthplace and skin color. In general, admixture estimations detected here ranged closely to a previous report on Cubans (Fortes-Lima et al., 2018). An element derived from the population structure described may have pharmacogenetic implications. The European and African components together account for 95% of the genetic diversity in this sample, as compared to the 5% for Amerindian ancestry. Consequently, European and African ancestry will have considerably greater impact on the frequency distribution of variants with pharmacological relevance.

There is a strong relationship between admixture proportions and census categories in the Cuban population, as suggested by Marcheco-Teruel et al. (2014). In agreement with these data, the estimations of European ancestry detected here decreased from self-reported White to Black individuals, and the opposite trend was observed in regard to the African ancestry, while admixed individuals displayed intermediate values for both ancestry components. By place of birth, European ancestry was predominant in the Western and Central regions, while the Eastern region had the highest African contribution, followed by the Western and Central regions.

Genetic differentiation, as described by F_ST_ pairwise comparisons within the Cuban population, suggested that drug responses may vary individually by skin color categories and regions of the country, with admixture proportions being a key factor. In this line, geographic differences observed in 11 genotype frequencies could also be associated, at least in part, with admixture patterns geographically structured across the island. Estimates of African and European ancestry by birthplace were comparable to reported proportions (Fortes-Lima et al., 2018). The central region showed a genotype distribution similar to that of the Spanish population, while individuals from the Eastern and Western provinces showed intermediate frequencies relative to the parental populations. Eastern provinces had relatively higher African contributions (average 34%) than Western and Central provinces. Data were readily extended to the greater genetic differentiation found in pairwise F_ST_ comparisons of the Central vs. Eastern regions, showing six markers with moderate divergence.

F_ST_ pairwise comparisons with other populations indicated genetic similarity to Spanish and Latin American populations. However, interethnic variability was observed. *CYP1A1* rs1048943 (CYP1A1*2C) is greatly represented in Latin American populations (Amerindian ancestry), whereas in Africans and Europeans, its frequency is low (Pérez-Morales et al., 2008). Given the small Amerindian component estimated in Cubans, moderate divergence was expected in comparison to Latin American populations (Figure 1). The global allele distribution of rs776746 (CYP3A5*3) increased from 18% to 94% in African to European populations (Fairley et al., 2019), respectively. On average, the frequency of CYP3A5*3 is approximately 80% in Latin Americans, but it was found to be lower in this study (69%), compared to other reports on Chileans (76%) (Roco et al., 2012), Brazilians (73%) (Suarez-Kurtz et al., 2014), and admixed Mexicans (74%) (Fricke-Galindo et al., 2016). On the contrary, rs2740574 (CYP3A4*1B) is found in African populations due to a suggested selection factor against non-African populations involving vitamin D metabolism (Schirmer et al., 2006). The CYP3A4*1B frequency in Cubans was higher than in Ecuadorians (Sinués et al., 2008), admixed Mexicans (Gonzalez-Covarrubias et al., 2019), and Chileans (Roco et al., 2012), but similar to that in Brazilians (Rodrigues-Soares et al., 2018).

Previous studies have reported about CYP allele frequencies in Cubans (Llerena et al., 2014; Rodrigues‐Soares et al., 2020). In individuals with European ancestry, rs35742686 (CYP2D6*3) is found at a low frequency (Zhou et al., 2017), as well in Latin Americans (Naranjo et al., 2018), and it is virtually absent in Chileans (Roco et al., 2012). In agreement with these data, CYP2D6*3 was not detected in the present sample cohort. CYP2D6*10 is related to Asian ancestry but is also distinctive of African populations together with CYP2D6*17. However, a higher *CYP2D6* *10 allele frequency was reported in this study (18%) and Brazilians (Suarez-Kurtz et al., 2012a). The six variant alleles studied in the *CYP2C* subfamily ranged closely to reports on Brazilians (Suarez-Kurtz et al., 2012b) and CYP2C9*2 and *3 to Venezuelans (Flores-Gutiérrez et al., 2017). CYP2C19*3 is less frequent than CYP2C19*2 worldwide and has its highest frequency in Asians (Zhou et al., 2017). It is almost non-existent in Latin America (Suarez-Kurtz et al., 2012b), it is not present in Chileans (Roco et al., 2012), and it was also not detected in this Cuban cohort. One of the highest frequencies of CYP2C19*2 and *3 is found in Venezuela, associated with their Amerindian component (Castro de Guerra et al., 2013).

Frequency estimates of *EPHX1* rs2234922 and *AHR* rs2066853 are 8% and 15% in Mexicans (Pérez-Morales et al., 2011), respectively, and 8% for *AHR* rs2066853 in Brazilians (Abnet et al., 2007), whereas we observed lower frequencies for both SNVs. The estimated frequency of rs1045642 (*ABCB1*) in this study (37%, A allele) was intermediate to the frequencies reported for Spaniards (48%) and African Americans (16%) but similar to those observed in Brazilians (39%) (Scheiner et al., 2010) and in a previous Cuban study (36.5%), with comparable demographical variables (Rodeiro et al., 2022).

The frequency of the *GSTT1* null genotype in Cubans was closer to that observed in Spanish than to Africans (Kasthurinaidu et al., 2015), with an F_ST_ value in Cuba vs. Africa comparison near the moderate differentiation threshold (F_ST_ = 0.045). Results are consistent with those of other studies stating that the *GSTM1* null genotype is more frequent than *GSTT1* worldwide (Nakanishi et al., 2022). The prevalence of both deletions was similar to admixed Mexicans (Palma-Cano et al., 2017) and Chileans (Roco et al., 2012) but lower than in Brazilians (Magno et al., 2009) and Colombians (Ramírez et al., 2019).

Variants and genotypes (germline DNA) do not depend on disease status or evolution, in contrast to somatic mutations; thus, its clinical use can be considered with strong confidence as predictive biomarkers. Several SNVs in genes related to DNA repair processes and growth factors have clinical annotations on PharmGKB with different levels of evidence (Whirl‐Carrillo et al., 2021). Therefore, validating the usefulness of the recommended markers would promote a safe and cost-effective use of drugs in the clinical management of patients.

To illustrate this statement, 23% of the global population carries the *EGFR* rs712829 T allele. Similarly, the frequency for Hispanics is 21%, with notable ethnic variations. This variant is less common in Peruvians, but reaches 28% in Puerto Ricans (Fairley et al., 2019) and 23% in Mexicans (Torres-Jasso et al., 2015). A higher frequency was observed in Cubans (32%), closer to the frequencies found in Europeans and Iberians. Regarding *VEGF* rs3025039, Hispanics have the highest frequency for the T variant allele, particularly among Mexicans and Peruvians. However, these variants were less common in the Cuban sample, and a low frequency is also observed in Iberians, Colombians (Fairley et al., 2019), and Brazilians (Carvalho et al., 2021). This is important in the context of cancer susceptibility, side effects, and response rate to *EGFR* and *VEGF*-targeting drugs. The variant allele of *EGFR* correlates with increased receptor expression (Heist and Christiani, 2009), whereas the *VEGF* variant is associated with significantly lower *VEGF* plasma levels (Sibertin-Blanc et al., 2015), which would make carriers suitable for *EGFR*-target therapies and less appropriate for *VEGF*-target therapies. Meanwhile, moderate differentiation to Africans was observed for rs1042522 (*TP53*), which had a 63% prevalence in Cubans. Studies in Latin America agree on the predominance of the *TP53* Arg72 allele (Rodrigues-Soares et al., 2018; Olloquequi et al., 2022). Few variants in DNA repair enzymes have been characterized in Latin American populations. The frequency of rs13181 (*ERCC2*), rs861539 (*XRCC3*), and rs25487 (*XRCC1*) in Cubans resembled those of Mexicans (Pérez-Morales et al., 2011) and Brazilians (Duarte et al., 2005).

Attention has been drawn to variants in methylation enzymes or methyl recognition binding proteins (Majchrzak-Celińska et al., 2019). SNVs in epigenetic writer–reader–eraser enzymes had noticeable differences in prevalence among Africans, Europeans (Iberians), and Latin Americans. Hispanics have the highest frequency of *MBD5* rs16828708 (Fairley et al., 2019). Its frequency in Cubans matched that of the Spanish, displaying moderate divergence from Latin Americans (Figure 1). Allele variants in *MBD2*/*4* and *EZH1*/*2* enzymes showed moderate differentiation in F_ST_ pairwise comparison to Africa. According to the 1000 Genomes Project database, the frequencies observed here are closer to Spaniards than to Africans. The *MGMT* enzyme is of special interest; its expression is ubiquitous in normal cells, with considerable variation in the activity in the same tissue of different individuals, and it is frequently overexpressed in tumors vs. non-neoplastic tissues (Christmann et al., 2011). Genome-wide association studies have revealed that non-coding SNVs of *MGMT* affect gene expression (Huang et al., 2018). The F_ST_ values indicated low divergence from Spain and Latin America, but several markers showed large-to-moderate genetic differentiation to Africans. The F_ST_ statistic provides an approach to estimating the genetic divergence of pharmacogenetic variants and its potential impact on clinical response to drugs in different populations or strata within a population (Suarez-Kurtz and de Araújo, 2022). It also supports that the distribution of genetic variants among Cubans is influenced by the large variability in ancestry proportions.

The cost-effectiveness of pharmacogenetic testing has been demonstrated, supporting its integration into clinical practice (Morris et al., 2022; Plumpton et al., 2019). The pharmacogenes described here have been widely studied for their usefulness in predicting and tailoring efficient therapeutic responses (Zhao et al., 2021) and have clinical annotations on the PharmGKB Knowledge Base (Altman, 2007), with variable levels of evidence for current clinical guidelines. The prevalence of variants in these pharmacogenes in the Cuban population justifies the implementation of pharmacogenetic tools. While the use of AIM panels to infer ancestry in pharmacogenetic studies is recommended, the use of descriptive variables like skin color categories and region of birth may also be informative in different contexts. For instance, it may explain how admixture diversity may be translated into different prevalence of genetic variants among skin color categories and country regions. This information should be taken into account when designing clinical trials and public health policies related to pharmacogenetic testing. As an illustrative example, important pharmacogenetic variants, such as CYP3A4*1B and CYP3A5*3, related to immunosuppressive drug responses, were found to have high divergence between self-identified White/Black individuals and between Central and Eastern provinces. Among the human *CYP3A* enzymes, *CYP3A4* and *CYP3A5* are considered the most important in drug metabolism. Approximately half of the medications that are metabolized by P450 are *CYP3A* substrates (Saiz-Rodríguez et al., 2020). Thus, the present results provided evidence for its applications in public health priorities across the island. A study limitation is that recruitment was most successful in Western provinces, mainly in Havana. Future comprehensive studies should promote the participation of individuals from all provinces in the Central and Eastern regions.

In summary, this data was a contribution to pharmacogenetic characterization of Cuban population and to the development of precision medicine in the country.

## Data Availability

The datasets presented in this article are not readily available because the nature of this research contains information that could compromise the participants’ privacy, and they did not agree to share their data publicly. Requests to access the datasets should be directed to the corresponding author IR-G.
