# Supplementary material for: Prevalence of single-nucleotide variants in twenty-five pharmacogenes from a Cuban sample cohort
Source: Front Pharmacol. 2024 Sep 27;15:1467036. doi: 10.3389/fphar.2024.1467036 (PMC11472837; doi:10.3389/fphar.2024.1467036)
Supplement: Supplementary file 1 [file Table1.docx]

Supplementary Material

| **Supplementary Table 1. General information about the genes and variants of a single nucleotide considered in present study and population minor allele frequencies** | | | | | | |
| --- | --- | --- | --- | --- | --- | --- |
| **Function** | **Gene** | **rs Number** | **alleles** | **DNA Change** | **Effect** | **Population MAF** |
| **Phase I metabolism** | *AHR* | rs2066853 | G/A | c.1661G>A | p.Arg554Lys | **0.27** (A) |
|  | *CYP1A1* | rs1048943 | T/C; CYP1A1*2C | c.1384A>G | p.Ile462Val | **0.13** (C) |
|  |  | rs1799814 | G/T; CYP1A1*4 | c.1382C>A | p.Thr461Asn | **0.01** (T) |
|  | *CYP2D6* | rs1065852 | G/A; CYP2D6*10 | c.100C>T | p.Pro34Ser | **0.24** (A) |
|  |  | rs28371706 | G/A; CYP2D6*17 | c.320C>T | p.Thr107Ile | **0.06** (A) |
|  |  | rs35742686 | T/-; CYP2D6*3 | c.775del (T>delT) | p.Arg259Glyfs Ter2 (frameshift) | **0.01 (-)** |
|  | *CYP2C8* | rs10509681 | T/C; CYP2C8*3 | c.1196A>G | p.Lys399Arg | **0.05** (C) |
|  |  | rs11572103 | T/A; CYP2C8*2 | c.805A>T | p.Ile269Phe | **0.05** (A) |
|  | *CYP2C9* | rs1057910 | A/C; CYP2C9*3 | c.1075A>C | p.Ile359Leu | **0.05** (C) |
|  |  | rs1799853 | C/T; CYP2C9*2 | c.430C>T | p.Arg144Cys | **0.05** (T) |
|  | *CYP3A4* | rs2242480 | C/T | c.1026+12 G>A | Intron variant | **0.42** (T) |
|  |  | rs2740574 | T/C; CYP3A4*1B (C) | g.99382096C>; 392A>G | Intergenic variant located in the 5-prime promoter region | **0.23** (C) |
|  | *CYP3A5* | rs776746 | T/C; CYP3A5*3 | c.219-237A>G; 6986A>G | Splicing defect (which results in a premature Ter codon, hence a non-functional protein) | **0.38** (T) |
|  | *CYP2C19* | rs4986893 | G/A; CYP2C19*3 | c.636G>A | p.Trp212Ter | **0.01** (A) |
|  |  | rs4244285 | G/A; CYP2C19*2 | c.681G>A | p.Pro227= (aberrant splice site, which results in a non-functional protein) | **0.22** (A) |
|  | *EPHX1* | rs2234922 | A/G | c.416A>G | p.His139Arg | **0.22** (G) |
| **Phase II metabolism** | *GSTM1* | NA | GSTM1*0; null | NA | Whole gene deletion | **0.11 – 0.67*** |
|  | *GSTT1* | NA | GSTT1*0; null | NA |  | **0.10 – 0.51*** |
| **Transporter** | *ABCB1* | rs1045642 | G/A | c.3435C>T | p.Ile1145= (No sequence alteration) | **0.40** (A) |
| **Transcription Factor** | *TP53* | rs1042522 | G/C | c.215C>G | p.Pro72Arg | **0.46** (G) |
| **Nucleotide Excision Repair** | *ERCC2* | rs13181 | T/G | c.2251A>C | p.Lys751Gln | **0.24** (G) |
| **Base Excision Repair** | *XRCC1* | rs25487 | T/C | c.1196A>G | p.Gln399Arg | **0.26** (T) |
| **Homologous Recombination** | *XRCC3* | rs861539 | G/A | c.722C>T | p.Thr241Met | **0.22** (A) |
| **DNA direct reversal repair** | *MGMT* | rs1762429 | C/T | c.81+8147C>T | intron variant | **0.48** (T) |
|  |  | rs1762438 | C/T | g.131263457C>T | intergenic variant | **0.47** (C) |
|  |  | rs4751104 | G/A | c.218+70717G>A | intron variant | **0.33** (A) |
|  |  | rs4751115 | T/C | c.367+3986T>C | intron variant | **0.39** (T) |
|  |  | rs7068306 | C/G | c.219-36122C>G | intron variant | **0.35** (G) |
|  |  | rs10764896 | G/A | c.219-34437G>A | intron variant | **0.40** (G) |
|  |  | rs11016798 | C/T | g.131256585C>T | intergenic variant, upstream MGMT gene | **0.41** (T) |
|  |  | rs11016879 | A/G | c.219-16377A>G | intron variant | **0.35** (A) |
|  |  | rs11016885 | T/C | c.367+4924T>C | intron variant | **0.26** (C) |
|  |  | rs12259379 | G/T | c.82-31165G>T | intron variant | **0.17** (T) |
|  |  | rs12763287 | T/G | c.367+7527T>G | non coding transcript exon variant; intron variant | **0.08** (G) |
| **Growth Factors (susceptibility / treatment response)** | *EGFR* | rs712829 | G/T | c.-216G>T | 5 prime UTR variant | **0.23** (T) |
|  | *VEGFA* | rs3025039 | C/T | c.*237C>T; +936C>T | 3 prime UTR variant | **0.13** (T) |
| **DNA methylation (susceptibility / treatment response)** | *MTHFR* | rs11121832 | T/C | c.586+149A>G | Non coding transcript exon variant; intron variant | **0.24** (T) |
|  | *EZH1* | rs4792953 | T/C | c.461-836A>G | Intron variant | **0.34** (T) |
|  |  | rs7359598 | T/C | g.42745452T>C | 2KB upstream variant; regulatory region variant | **0.38** (T) |
|  | *EZH2* | rs2072408 | A/G | c.1947+520T>C | Intron variant | **0.24** (A) |
|  | *MBD2* | rs603097 | G/A | g.54226736G>A | intergenic variant | **0.11** (G) |
|  | *MBD4* | rs140695 | T/C | c.1412-54A>G | Intron variant/ non coding transcript exon variant | **0.15** (T) |
|  | *MBD5* | rs16828708 | A/G | c.*200A>G | 3 prime UTR variant | **0.45** (G) |
| Genome Assembly GRCh38 (26). MAF: Minor Allele Frequencies. MAF data from 1000 Genomes Project (22), *reference paper for GST null alleles (46). | | | | | | |

**Supplementary Table 2. Autosomal Ancestry Informative Markers (AIMs) genotyped in the study.**

| **Chromosome** | **Position in pb (hg38)** | **Locus Name (SNV ID)** | **Gene** |
| --- | --- | --- | --- |
| 1 | 36302599 | rs1573020 | THRAP3 |
|  | 159204893 | rs2814778 | ACKR1 |
|  | 204821849 | rs2065160 | ARHGEF1 |
| 2 | 7009024 | rs896788 | RNF144A |
|  | 135859184 | rs182549 | MCM6 |
| 3 | 168927247 | rs1498444 | intergenic variant |
| 4 | 38801634 | rs4540055 | TLR1 |
|  | 158260811 | rs2026721 | regulatory region |
| 5 | 33951588 | rs16891982 | SLC45A2 |
| 6 | 164624301 | rs727811 | intergenic variant |
| 7 | 4417372 | rs917118 | intergenic variant |
| 10 | 17022993 | rs7897550 | CUBN |
|  | 34466420 | rs1978806 | PARD3 |
| 11 | 32402843 | rs5030240 | WT1 |
| 12 | 29216938 | rs10843344 | FAR2 |
|  | 56210050 | rs773658 | RNF41 |
| 13 | 20327585 | rs1335873 | intergenic variant |
|  | 21800561 | rs1886510 | intergenic variant |
|  | 34290103 | rs2065982 | intron variant |
| 14 | 35701401 | rs10141763 | RALGAPA1 |
|  | 100676553 | rs730570 | intergenic variant |
| 15 | 28120472 | rs12913832 | HERC2 |
|  | 48134287 | rs1426654 | SLC24A5 |
| 16 | 31068050 | rs881929 | ZNF668 |
|  | 90038925 | rs3785181 | GAS8 |
| 17 | 77555585 | rs2304925 | intergenic variant |
| 18 | 77720430 | rs1024116 | intergenic variant |
| 19 | 41906179 | rs2303798 | MIR99AHG |
| 20 | 40221002 | rs1321333 | intergenic variant |
| 21 | 15313279 | rs722098 | intergenic variant |
|  | 16338103 | rs239031 | regulatory region |
|  | 24300147 | rs2572307 | regulatory region |
| 22 | 25954137 | rs5997008 | MYO18B |
|  | 47440662 | rs2040411 | intergenic variant |
| AIMs Panel from reference paper (18) | | | |

**Supplementary Table 3. Pairwise Fst values across subgroups of skin color, region of origin and sex within Cuban population and compared to other populations.**

| **Gene** | **SNP** | **Fst (skin color)** | | | **Fst (region of origin)** | | | **Fst (sex)** | **Fst (population)** | | | |
| --- | --- | --- | --- | --- | --- | --- | --- | --- | --- | --- | --- | --- |
|  |  | (Admixed-Black) | (White-Admixed) | (White-Black) | (Center-Eastern) | (Western-Center) | (Western-Eastern) | (female-male) | (Cuba-World) | (Cuba-Spain) | (Cuba-Africa) | (Cuba-AMR) |
| *CYP1A1* | rs1048943 T>C | 0,033 | 0,003 | 0,019 | 0,008 | 0,031 | 0,058 | 0,001 | 0,007 | 0,019 | 0,042 | 0,108 |
|  | rs1799814 G>T | 0,003 | 0,002 | 0,01 | 0,002 | 0,002 | 0 | 0,001 | 0,009 | 0,007 | 0,02 | 0,001 |
| *CYP2D6* | rs1065852 G>A | 0,001 | 0,006 | 0,011 | 0,011 | 0,001 | 0,006 | 0 | 0,005 | 0 | 0,01 | 0,002 |
|  | rs28371706 G>A | 0,001 | 0,039 | 0,05 | 0,015 | 0,007 | 0,002 | 0,004 | 0 | 0,014 | 0,084 | 0,014 |
| *CYP2C8* | rs11572103 T>A | 0,009 | 0,019 | 0,049 | 0,029 | 0,014 | 0,004 | 0 | 0 | 0,019 | 0,043 | 0,019 |
|  | rs10509681 T>C | 0,006 | 0,004 | 0,02 | 0,008 | 0,015 | 0,001 | 0 | 0,009 | 0,006 | 0,053 | 0 |
| *CYP2C9* | rs1799853 C>T | 0,009 | 0,001 | 0,016 | 0,006 | 0,012 | 0,001 | 0 | 0,009 | 0,004 | 0,053 | 0 |
|  | rs1057910 A>C | 0,003 | 0,001 | 0,001 | 0,003 | 0,003 | 0 | 0,001 | 0,003 | 0,012 | 0,012 | 0,001 |
| *CYP3A4* | rs2242480 C>T | 0,026 | 0,074 | 0,177 | 0,057 | 0,013 | 0,016 | 0,002 | 0,009 | 0,063 | 0,279 | 0,004 |
|  | rs2740574 T>C | 0,013 | 0,093 | 0,164 | 0,06 | 0,036 | 0,003 | 0,002 | 0 | 0,088 | 0,281 | 0,026 |
| *CYP3A5* | rs776746 T>C | 0,044 | 0,071 | 0,214 | 0,063 | 0,036 | 0,004 | 0,004 | 0,005 | 0,094 | 0,276 | 0,016 |
| *CYP2C19* | rs4244285 G>A | 0,019 | 0,012 | 0,001 | 0,001 | 0 | 0,001 | 0,002 | 0,014 | 0,001 | 0,003 | 0,001 |
| *EPHX1* | rs2234922 A>G | 0,013 | 0,003 | 0,027 | 0,006 | 0,004 | 0 | 0,001 | 0 | 0,008 | 0,051 | 0,008 |
| *AHR* | rs2066853 G>A | 0,032 | 0,023 | 0,105 | 0,02 | 0,02 | 0 | 0 | 0,003 | 0,018 | 0,059 | 0,004 |
| *ABCB1* | rs1045642 G>A | 0 | 0,015 | 0,018 | 0,02 | 0,012 | 0,001 | 0 | 0,001 | 0,008 | 0,077 | 0,004 |
| *MBD4* | rs140695 T>C | 0,003 | 0,024 | 0,044 | 0,049 | 0,01 | 0,016 | 0,001 | 0,008 | 0,008 | 0,095 | 0,001 |
| *MBD2* | rs603097 G>A | 0,003 | 0,008 | 0,02 | 0,005 | 0,013 | 0,002 | 0,001 | 0,001 | 0,005 | 0,07 | 0,017 |
| *MTHFR* | rs11121832 T>C | 0,017 | 0,001 | 0,009 | 0 | 0 | 0,001 | 0 | 0,002 | 0,007 | 0 | 0,021 |
| *MBD5* | rs16828708 A>G | 0 | 0 | 0 | 0,001 | 0,003 | 0 | 0,001 | 0,027 | 0 | 0 | 0,06 |
| *EZH2* | rs2072408 A>G | 0,007 | 0,014 | 0,04 | 0,014 | 0,005 | 0,002 | 0,009 | 0,001 | 0,008 | 0,124 | 0,001 |
| *EZH1* | rs4792953 T>C | 0,017 | 0,034 | 0,096 | 0,027 | 0,003 | 0,013 | 0,005 | 0,001 | 0,007 | 0,131 | 0,005 |
|  | rs7359598 T>C | 0,017 | 0,033 | 0,094 | 0,023 | 0,004 | 0,008 | 0,006 | 0,004 | 0,004 | 0,127 | 0,014 |
| *VEGF* | rs3025039 C>T | 0 | 0 | 0,001 | 0,004 | 0,001 | 0,001 | 0,001 | 0 | 0 | 0,007 | 0,021 |
| *EGFR* | rs712829 G>T | 0 | 0,035 | 0,03 | 0,032 | 0,001 | 0,044 | 0 | 0,01 | 0 | 0 | 0,016 |
| *TP53* | rs1042522 G>C | 0,026 | 0,011 | 0,069 | 0,035 | 0,043 | 0 | 0,001 | 0,008 | 0,005 | 0,073 | 0,003 |
| *ERCC2* | rs13181 T>G | 0 | 0 | 0 | 0 | 0,001 | 0 | 0,001 | 0,003 | 0 | 0,017 | 0,006 |
| *XRCC1* | rs25487 T>C | 0,021 | 0,001 | 0,031 | 0 | 0 | 0,001 | 0,002 | 0 | 0,025 | 0,042 | 0,002 |
| *XRCC3* | rs861539 G>A | 0,024 | 0,004 | 0,046 | 0,025 | 0,013 | 0,002 | 0,002 | 0,01 | 0,009 | 0,019 | 0,008 |
| *MGMT* | rs10764896 G>A | 0 | 0 | 0 | 0,006 | 0,004 | 0 | 0 | 0,002 | 0,005 | 0,005 | 0,005 |
|  | rs11016798 C>T | 0,037 | 0,008 | 0,078 | 0,048 | 0,026 | 0,004 | 0,005 | 0,002 | 0,017 | 0,219 | 0,004 |
|  | rs11016879 A>G | 0,01 | 0,005 | 0,03 | 0,002 | 0,005 | 0,001 | 0 | 0 | 0 | 0,049 | 0,011 |
|  | rs11016885 T>C | 0,031 | 0,027 | 0,107 | 0,023 | 0,009 | 0,003 | 0,003 | 0,001 | 0,006 | 0,107 | 0,005 |
|  | rs12259379 G>T | 0,018 | 0,022 | 0,076 | 0,032 | 0,017 | 0,003 | 0,003 | 0 | 0,028 | 0,114 | 0,003 |
|  | rs4751104 G>A | 0,001 | 0,004 | 0,001 | 0,003 | 0,002 | 0 | 0,01 | 0,009 | 0,011 | 0,023 | 0 |
|  | rs12763287 T>G | 0,016 | 0 | 0,016 | 0 | 0,001 | 0 | 0,001 | 0,003 | 0,002 | 0,044 | 0,003 |
|  | rs1762429 C>T | 0,052 | 0,008 | 0,099 | 0,049 | 0,026 | 0,004 | 0,005 | 0 | 0,02 | 0,206 | 0,004 |
|  | rs1762438 C>T | 0,01 | 0,002 | 0,02 | 0,01 | 0,012 | 0 | 0,003 | 0 | 0,006 | 0,01 | 0 |
|  | rs4751115 T>C | 0,009 | 0,003 | 0,022 | 0,007 | 0,002 | 0,002 | 0 | 0 | 0,017 | 0,017 | 0,008 |
|  | rs7068306 C>G | 0,006 | 0,002 | 0,015 | 0,004 | 0,002 | 0 | 0 | 0,002 | 0,001 | 0,027 | 0,004 |
| *GSTM1* | null | 0 | 0,011 | 0,011 | 0 | 0 | 0 | 0 | 0,005 | 0,012 | 0,009 | 0,003 |
| *GSTT1* |  | 0,01 | 0 | 0,012 | 0,008 | 0,008 | 0 | 0,001 | 0,009 | 0,003 | 0,045 | 0,007 |
| Cuba N=357, White=190, Admixed=101, Black=66, Western=174, Center=49, Eastern=134. Population data from 1000 Genome Project (22): World N=2504, Spain N=107, African N=661, AMR (Latin American) N=347. | | | | | | | | | | | | |

**Supplementary Table 4. Alternative allele frequencies of the studied loci in the Cuban general population, stratified by skin color of individuals, region of origin and sex. Frequency reports across different populations are shown.**

| **Gene** | **SNV** | **CUBA** | | | | | | | | | **EUR** | **AFR** | **AMR** | **World** |
| --- | --- | --- | --- | --- | --- | --- | --- | --- | --- | --- | --- | --- | --- | --- |
|  |  | total | White | Admixed | Black | Western | Center | Eastern | female | male | Iberian | all | all | all |
|  |  | (357) | (190) | (101) | (66) | (174) | (49) | (134) | (188) | (169) | (107) | (661) | (347) | (2504) |
| *CYP1A1* | rs1048943 T>C | 0,08 | 0,08 | 0,11 | 0,02 | 0 | 0,06 | 0,11 | 0,09 | 0,07 | 0,02 | 0,01 | 0,35 | 0,13 |
|  | rs1799814 G>T | 0,04 | 0,06 | 0,04 | 0,02 | 0,04 | 0,06 | 0,04 | 0,05 | 0,04 | 0,08 | <0,01 | 0,03 | 0,01 |
| *CYP2D6* | rs1065852 G>A | 0,18 | 0,21 | 0,15 | 0,13 | 0,2 | 0,22 | 0,14 | 0,18 | 0,17 | 0,17 | 0,11 | 0,15 | 0,24 |
|  | rs28371706 G>A | 0,05 | 0,01 | 0,1 | 0,12 | 0,05 | 0,02 | 0,07 | 0,04 | 0,07 | 0,01 | 0,22 | 0,01 | 0,06 |
| *CYP2C8* | rs11572103 T>A | 0,06 | 0,02 | 0,08 | 0,14 | 0,05 | 0,01 | 0,08 | 0,06 | 0,06 | 0,01 | 0,19 | 0,01 | 0,05 |
|  | rs10509681 T>C | 0,1 | 0,13 | 0,09 | 0,05 | 0,08 | 0,16 | 0,1 | 0,11 | 0,1 | 0,15 | 0,01 | 0,1 | 0,05 |
| *CYP2C9* | rs1799853 C>T | 0,1 | 0,12 | 0,1 | 0,05 | 0,08 | 0,15 | 0,1 | 0,1 | 0,09 | 0,14 | 0,01 | 0,1 | 0,05 |
|  | rs1057910 A>C | 0,03 | 0,03 | 0,04 | 0,02 | 0,03 | 0,05 | 0,03 | 0,04 | 0,03 | 0,08 | <0,01 | 0,04 | 0,05 |
| *CYP3A4* | rs2242480 C>T | 0,33 | 0,18 | 0,43 | 0,59 | 0,3 | 0,2 | 0,42 | 0,31 | 0,35 | 0,12 | 0,85 | 0,39 | 0,42 |
|  | rs2740574 T>C | 0,23 | 0,09 | 0,34 | 0,45 | 0,23 | 0,09 | 0,28 | 0,21 | 0,25 | 0,03 | 0,77 | 0,11 | 0,23 |
| *CYP3A5* | rs776746 T>C | 0,69 | 0,84 | 0,6 | 0,39 | 0,69 | 0,85 | 0,63 | 0,72 | 0,66 | 0,93 | 0,18 | 0,8 | 0,62 |
| *CYP2C19* | rs4244285 G>A | 0,13 | 0,15 | 0,08 | 0,17 | 0,14 | 0,14 | 0,12 | 0,12 | 0,15 | 0,15 | 0,17 | 0,11 | 0,22 |
| *EPHX1* | rs2234922 A>G | 0,21 | 0,17 | 0,21 | 0,31 | 0,21 | 0,16 | 0,22 | 0,2 | 0,22 | 0,14 | 0,35 | 0,14 | 0,22 |
| *AHR* | rs2066853 G>A | 0,22 | 0,13 | 0,25 | 0,42 | 0,24 | 0,13 | 0,24 | 0,22 | 0,23 | 0,12 | 0,46 | 0,17 | 0,27 |
| *ABCB1* | rs1045642 G>A | 0,37 | 0,43 | 0,31 | 0,3 | 0,37 | 0,48 | 0,34 | 0,37 | 0,38 | 0,46 | 0,15 | 0,43 | 0,4 |
| *MBD4* | rs140695 T>C | 0,78 | 0,71 | 0,84 | 0,88 | 0,75 | 0,66 | 0,85 | 0,77 | 0,79 | 0,7 | 0,98 | 0,81 | 0,85 |
| *MBD2* | rs603097 G>A | 0,87 | 0,84 | 0,9 | 0,93 | 0,89 | 0,81 | 0,86 | 0,86 | 0,88 | 0,82 | 0,99 | 0,77 | 0,89 |
| *MTHFR* | rs11121832 T>C | 0,72 | 0,73 | 0,76 | 0,64 | 0,71 | 0,73 | 0,74 | 0,72 | 0,72 | 0,79 | 0,68 | 0,84 | 0,76 |
| *MBD5* | rs16828708 A>G | 0,29 | 0,3 | 0,28 | 0,29 | 0,28 | 0,33 | 0,3 | 0,31 | 0,28 | 0,29 | 0,26 | 0,53 | 0,45 |
| *EZH2* | rs2072408 A>G | 0,78 | 0,72 | 0,82 | 0,88 | 0,77 | 0,71 | 0,81 | 0,74 | 0,82 | 0,7 | 0,97 | 0,8 | 0,76 |
| *EZH1* | rs4792953 T>C | 0,63 | 0,53 | 0,71 | 0,82 | 0,6 | 0,55 | 0,71 | 0,6 | 0,67 | 0,55 | 0,93 | 0,56 | 0,66 |
|  | rs7359598 T>C | 0,56 | 0,45 | 0,63 | 0,75 | 0,53 | 0,47 | 0,62 | 0,52 | 0,6 | 0,5 | 0,87 | 0,44 | 0,62 |
| *VEGF* | rs3025039 C>T | 0,12 | 0,11 | 0,12 | 0,13 | 0,11 | 0,09 | 0,13 | 0,11 | 0,13 | 0,13 | 0,08 | 0,23 | 0,13 |
| *EGFR* | rs712829 G>T | 0,32 | 0,38 | 0,21 | 0,22 | 0,38 | 0,35 | 0,19 | 0,32 | 0,32 | 0,33 | 0,25 | 0,21 | 0,23 |
| *TP53* | rs1042522 G>C | 0,63 | 0,71 | 0,61 | 0,45 | 0,6 | 0,79 | 0,62 | 0,65 | 0,62 | 0,7 | 0,33 | 0,68 | 0,54 |
| *ERCC2* | rs13181 T>G | 0,29 | 0,3 | 0,28 | 0,28 | 0,28 | 0,31 | 0,3 | 0,28 | 0,31 | 0,31 | 0,19 | 0,22 | 0,24 |
| *XRCC1* | rs25487 T>C | 0,73 | 0,69 | 0,72 | 0,84 | 0,72 | 0,73 | 0,74 | 0,71 | 0,75 | 0,58 | 0,89 | 0,69 | 0,74 |
| *XRCC3* | rs861539 G>A | 0,31 | 0,36 | 0,3 | 0,17 | 0,31 | 0,42 | 0,27 | 0,33 | 0,29 | 0,4 | 0,19 | 0,23 | 0,22 |
| *MGMT* | rs10764896 G>A | 0,56 | 0,56 | 0,57 | 0,55 | 0,56 | 0,5 | 0,58 | 0,57 | 0,55 | 0,49 | 0,63 | 0,49 | 0,6 |
|  | rs11016798 C>T | 0,44 | 0,51 | 0,42 | 0,24 | 0,44 | 0,6 | 0,38 | 0,47 | 0,4 | 0,57 | 0,12 | 0,5 | 0,4 |
|  | rs11016879 A>G | 0,66 | 0,61 | 0,68 | 0,77 | 0,68 | 0,61 | 0,65 | 0,66 | 0,66 | 0,67 | 0,85 | 0,56 | 0,65 |
|  | rs11016885 T>C | 0,28 | 0,38 | 0,23 | 0,1 | 0,29 | 0,38 | 0,24 | 0,31 | 0,26 | 0,35 | 0,07 | 0,22 | 0,25 |
|  | rs12259379 G>T | 0,18 | 0,11 | 0,22 | 0,34 | 0,18 | 0,09 | 0,22 | 0,16 | 0,2 | 0,07 | 0,42 | 0,14 | 0,18 |
|  | rs4751104 G>A | 0,42 | 0,4 | 0,46 | 0,43 | 0,42 | 0,38 | 0,43 | 0,37 | 0,47 | 0,32 | 0,52 | 0,42 | 0,33 |
|  | rs12763287 T>G | 0,11 | 0,12 | 0,12 | 0,05 | 0,1 | 0,12 | 0,11 | 0,12 | 0,1 | 0,14 | 0,01 | 0,08 | 0,08 |
|  | rs1762429 C>T | 0,49 | 0,57 | 0,48 | 0,26 | 0,49 | 0,65 | 0,43 | 0,52 | 0,45 | 0,63 | 0,14 | 0,55 | 0,47 |
|  | rs1762438 C>T | 0,51 | 0,54 | 0,5 | 0,4 | 0,49 | 0,6 | 0,5 | 0,53 | 0,48 | 0,59 | 0,42 | 0,51 | 0,53 |
|  | rs4751115 T>C | 0,62 | 0,58 | 0,63 | 0,72 | 0,61 | 0,57 | 0,65 | 0,63 | 0,62 | 0,49 | 0,75 | 0,53 | 0,61 |
|  | rs7068306 C>G | 0,31 | 0,34 | 0,3 | 0,23 | 0,31 | 0,27 | 0,33 | 0,3 | 0,32 | 0,34 | 0,18 | 0,37 | 0,35 |
| *GSTM1* | null | 0,39 | 0,44 | 0,34 | 0,34 | 0,39 | 0,4 | 0,39 | 0,38 | 0,40 | 0,50 | 0,38 | 0,44 | 0,11-0,65 |
| *GSTT1* |  | 0,18 | 0,16 | 0,17 | 0,25 | 0,17 | 0,24 | 0,17 | 0,17 | 0,19 | 0,22 | 0,29 | 0,25 | 0,10-0,51 |
| EUR: European, AFR: African, AMR: American. Sample size for each column is located inside parenthesis. Frequencies were collected from 1000 Genome Project (22), except for *GSTM1* and *GSTT1* where frequencies were extracted from (48) for AFR and AMR and from (47) for Iberians and the World. | | | | | | | | | | | | | | |
